# Connecting the nervous and the immune systems in evolution

**DOI:** 10.1038/s42003-018-0070-2

**Published:** 2018-06-07

**Authors:** Volker Hartenstein, Angela Giangrande

**Affiliations:** 1 Department of Molecular, Cell and Developmental Biology, University of California Los Angeles, Los Angeles, CA 90095-1606 USA; 2 Institut de Génétique et de Biologie Moléculaire et CellulaireI, IGBMC/CNRS/INSERM/UDS, BP 10142, 67404 ILLKIRCH, CU de Strasbourg, France

## Abstract

Despite their great importance for biomedical research, the intricate network of relationships between macro- and microglia, in terms of development, function and evolution, remains poorly understood. Drawing inspiration from the recent meeting “Of Glia and Microglia”, held at the University of Strasbourg in December 2017, we here discuss the outstanding questions in the seemingly disparate fields of glial development, physiology and evolution, and also provide suggestions for how the field should move forward.

## Abstract (web summary)

Volker Hartenstein and Angela Giangrande discuss recent advances and future directions in glial biology and evolution in the context of a recent scientific conference. Their Comment illustrates the importance of interdisciplinary approaches to answering outstanding questions in biology.

## Introduction

Glial cells ensure normal brain structure and function. They are also involved in a broad range of pathological conditions, including brain injury, acute infection, autoimmune disorders and cancer. This cell population has been historically subdivided into macroglia and microglia, even though it is now clear the two cells types have different origins and properties. Within the Central Nervous System (CNS), macroglia and microglia regulate many neural functions that span the range from development, function and immune response^1,2^. Macroglia include prominent cell types such as astrocytes and oligodendrocytes which, like neurons, are neurectodermally derived lineages. Macroglia allow the neurons to develop and migrate. They also insulate and protect neurons and axons. Depending on the developmental stage and on environmental conditions, astrocytes can also display neural stem cell or phagocytic properties^3,4^. Microglia, on the other hand, are mesoderm-derived immune cells that invade the CNS during early development. They constitute the professional scavenger cells in physiological and pathological conditions. Thus, the most parsimonious hypothesis is that glial cells are simply non-neuronal cells that take on specific functions and markers.

To improve our capability to manage pathologies affecting the human nervous system, we must develop a more complete picture of the shared molecular and genetic pathways active during the development and function of macroglia and microglia. Towards that end, it is important to understand the evolutionary origin of these cells and how they function in other species, as this will further inform our understanding of these systems and aid in the development of models for experimental investigation and validation. The recent meeting in Strasbourg entitled “Of Glia and Microglia” (https://www.neurex.org/events/archives/item/22-of-glia-and-microglia) gathered scientists at the frontline of research into glia and phagocyte biology, in both vertebrate and invertebrate systems. The meeting was supported by Neurex, an European network that federates neuroscientists within the Universities of Strasbourg (France), Basel (Switzerland) and Freiburg (Germany), and sponsored by Leica. The event helped outline cross-disciplinary approaches that treat the questions of how glial cells differentiate (development), work (physiology) and why they work in that way (evolution) as indivisible objectives. Here we discuss the main themes of the meeting, including the role of these cell types in neural function and pathology. We also provide some suggestions for future directions in this research area.

## Glial development in vertebrates and *Drosophila*

The neuroectodermal origin of vertebrate macroglial cells was demonstrated many decades ago. More recently, it has been shown that microglia originate from mesodermal embryonic blood progenitors and migrate into the nervous system during development^5^. Many invertebrates also possess neuroectodermally-derived macroglial cells. These can be divided into several classes of cells: those that cover the surface of the nervous system; those that envelop neuronal cell bodies; and glia associated with the neuropile^6^. It is not clear whether vertebrate and invertebrate macroglia are truly homologous, i.e., derived from a primordial glial cell that was already present in the common bilaterian ancestor^7^. In addition, the presence of distinct immune-active microglia among invertebrates is uncertain, with the notable exception of annelids, and specific invertebrate macroglia also display a microglia-like immune function (see below). Finally, blood-derived macrophages cooperate with macroglial cells during invertebrate nervous system development and restructuring^8^. Altogether, these findings highlight the tight connection between immune and neural development.

Microglial development has made the object of intense investigation. In the classical mammalian mononuclear phagocyte system, as Dr. Elisa Gomez Perdiguero (Institut Pasteur, Paris) discussed at the meeting, it has been assumed that resident macrophages, including microglia, are derived and constantly replenished by circulating monocytes. However, recent research points to an origin during early embryogenesis^5,9^. Specifically, microglia can be traced to a population of multipotent progenitors that arise from the yolk sac during an interval bridging primitive and definitive hematopoiesis.

The formation of microglia also differs among vertebrates. The process in Zebrafish exhibits many similarities to that in mammals, but also shows significant idiosyncrasies. Importantly, their origin and spreading can be directly followed in the fast developing fish embryo^10^. Dr. Philippe Herbomel (Institut Pasteur, Paris) visualized the dynamic changes in form, phagocytic behavior and marker expression of developing macrophages to show that from their first arrival in the cephalic mesenchyme, primitive macrophages engulf apoptotic neurons. Interestingly, during metamorphosis to adult zebrafish, embryonically developed microglia are replaced by macrophages derived from definitive hematopoietic stem cells (HSCs), a phenomenon that distinguishes fish from mammals.

Microglia have so far not been described for the fruit fly *Drosophila melanogaster*. In this organism, as noted by several speakers, neuroectodermally derived cells carry out many immune functions in the CNS, suggesting that a single cell type carries out the role of macro- and microglia. While these cells are generally called glia and all express the pan-glial marker Repo, different subtypes seem to accomplish specific functions. Dr. Estee Kurant (University of Haifa) showed that the glial subtype enveloping the neuronal cell bodies also called cortex glia act as non-professional phagocytes clearing up apoptotic cells in the developing nervous system^11^. “Eat me” signals expressed by certain neurons activate phagocytic receptors, including Six Microns Under (SIMU) and Draper (DRPR) in those glia. Interestingly, overexpression of these receptors in adult fly brain glia evokes excessive neuronal loss in a process that might be comparable to phagoptosis, whereby macrophages or glia engulf live, stressed neurons. Phagoptosis has been recognized as an important mechanism of neurodegeneration^12^, which could be conserved from flies to vertebrates.

While glial cells provide the immune function within the *Drosophila* CNS, professional macrophages circulating in the body cavity carry out an immune function outside the nervous system. These macrophages are born during an early wave in the embryonic head mesoderm, followed by a second wave in the larval lymph gland. Comparable to tissue-resident macrophages of vertebrates, a significant number of early macrophages takes residence in segmental pockets underneath the body wall, which is formed by body wall muscles^13^ and by sensory neurons. These neurons, whose cell body is located just beneath the epidermis, play a key role for the adhesion and the proliferative activity of such semi-stationary blood cells. As noted by Dr. Katja Brückner (University of California, San Francisco), this could represent an adaptive mechanism that boosts the immune system of larvae encountering adverse environmental conditions.

There is still much to learn about invertebrate glial cells. As discussed by Dr. Tapio Heino (University of Helsinki), a novel cell type with many properties of microglia (MiC) can be induced cell non-autonomously by activating immune- related pathways in glia^14^. MiCs invade the neuropil and express structural and molecular markers of macrophages (Zfh1, Relish and the scavenger receptor Draper), though they do not express markers of glia or neurons. Their time of appearance and the expression of the midline marker Sim (Single minded) suggest that MiCs are descendants of midline cells, which have been thoroughly described for their role in axon guidance and neural morphogenesis in the embryo. Interestingly, midline cells originate from the mesectoderm, a population of cells at the border between the neuroectoderm and the mesoderm that normally undergoes apoptosis during postembryonic development. The discovery of the MiCs calls for a second type of immune cells in the CNS, in addition to the glial cells expressing Repo.

## Evolutionary origin

**Fig. 1:**
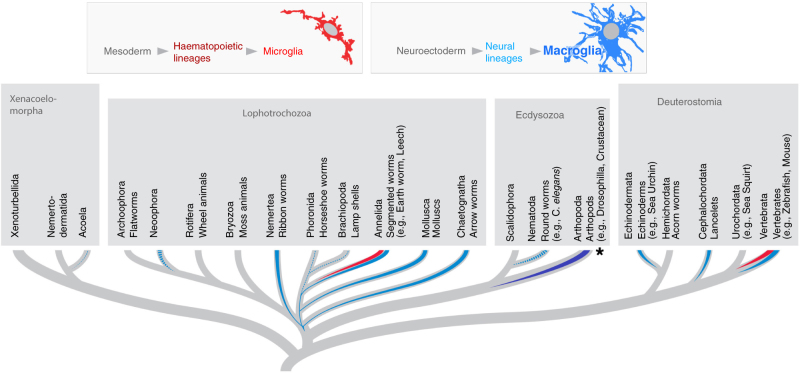
Macroglia and microglia in evolution. Schematic phylogenetic tree of Bliaterian animals (after Cannon et al.^7^) showing occurrence of neuroectodermally derived macroglia (blue) and mesodermally derived microglia (red). Note that, for most invertebrate taxa, no systematic analysis of microglia or other macrophages associated with the nervous system has been done yet. In annelids (leech), where microglia are abundant, the mesodermal origin needs to be ascertained. In at least some arthropods (e.g., *Drosophila*; asterisk and dark blue rendering of line), neurectodermally derived glia express genetic markers for and carry out the phagocytic function of microglia. Dashed lines indicate uncertainty regarding presence of glia

On the basis of the above data, we can then ask when, in evolution, do immune cells start invading the nervous system and acquire the role of professional scavengers, leading to cells with the distinct properties of microglia? The question of the evolutionary origin of glia cells requires an agreed upon definition of these cells. In the absence of conserved molecular markers, the classical definition of macroglia relies on the intimate association with neurons and the production of sheath-like processes that envelop neuronal somata and axons^6^. Using this definition, Dr. Daniel Hartline (University of Hawaii) discussed the occurrence of macroglia across the animal kingdom. Macroglia are absent in prebilaterians (Cnidaria, Ctenophora) and in numerous basal branches of the bilaterian superclades (Lophotrochozoa, Ecdysozoa, Deuterostomia), but are present in other groups of these superclades, notably molluscs and annelids, arthropods, and chordates (Fig. 1). Similarly, dedicated macrophages of the CNS resembling microglia are present in Annelida and in vertebrates but not in other clades. It is therefore likely that glia (macro and micro) evolved multiple times independently. This illustrates the importance of using a broader spectrum of model organisms for the study of glial biology. It also explains the somewhat heterogeneous nomenclature of the glial subtypes in different species. A clearer picture of glial evolution will only be available once the transcriptional landscape and lineage tracing are obtained for different cell types and organisms.

There are some initial findings pointing to the presence of macroglia in the ascidian, *Ciona intestinalis*, as reported by Dr. Marios Chatzigeorgiou (SARS, Bergen). With a candidate screen, Dr. Chatzigeorgiou and colleagues found that numerous vertebrate glial markers are expressed in the *Ciona* larva; several markers showed expression in close relationship to the larval cerebral vesicle and neural tube. The identification of functionally conserved macroglial cells will fill a gap in our understanding of the glial biology in deuterostomes.

The evolutionary relationship between macroglia and microglia/macrophages was highlighted by Dr. Volker Hartenstein (University of California, Los Angeles), who focused on glial development in the basal bilaterian clade Acoela (Fig. 1). They found an absence of specialized glial cell type, although there were many different acoel cell types that form lamelliform processes present. This supports the idea that glia present in bilaterian taxa with large, complex nervous systems have evolved convergently.

Building on this, Dr. Detlev Arendt (EMBL, Heidelberg) provided a conceptual background for understanding glial structure and function throughout phylogeny, within the broader context of an evolutionary framework for understanding the origin of cell types. These are defined by the expression of molecular complexes, termed apomeres, which are specialized for different functions, such as excretion, contraction or electrical conduction^15^. While combined in a single cell in the metazoan ancestor, these apomeres became segregated into different cell types during early metazoan evolution by means of differentially expressed transcriptional complexes, termed core regulatory networks.

In line with this, the cell type known as glia may represent a combination of separate glial apomeres specialized for different functions, including phagocytosis/immunity, neural progenitor, axonal enwrapment and transmitter homeostasis. In complex organisms one cell type may still recruit more than one apomere. For example, vertebrate astrocytes retain the engulfing machinery even after the appearance of microglial cells, which are considered to be the scavenger cells of the CNS. Understanding whether or not astrocytes and microglia are really functionally redundant would benefit of comparative transcriptome analyses in basal and challenged conditions. In simple organisms, however, it is clear that a single cell type performing several functions is a much more common feature, due to the reduced number of cell types. For example, a single cell type carries out the role of micro- and macroglia in the *Drosophila* nervous system.

Dr. Angela Giangrande (IGBMC, Strasbourg) showed that, in *Drosophila*, the transcription factor Glial cells missing (Gcm) is necessary in both embryonic glia and macrophages^16^. In glia, it induces the expression of the pan-glial homeodomain transcription factor Repo. In macrophages, it inhibits the Jak/Stat pathway, whose over-activation results in an enhanced inflammatory response. Thus, in line with the apomere hypothesis, the same developmental gene controls the cells with immune function, regardless of the cell origin. The role of the transiently expressed *gcm* gene in the inflammatory response also suggests that developmental pathways imprint a physiological status and hence affect late events, similar to what has been found in severe human pathologies of the nervous system (see below). Interestingly, the murine *gcm* orthologs are expressed in the immune system but not during gliogenesis, suggesting that the immune function may represent an ancestral feature of this molecular pathway.

## Glial function and possible implications for human health

Glial cells play crucial roles in the wiring and function of the vertebrate CNS and in the repair of CNS injury. As demonstrated by several speakers, there is now evidence that these pathways may be evolutionarily conserved.

Microglia interact with precursors of GABAergic inhibitory interneurons as they migrate from the ganglionic eminence into the cortex^17^. Dr. Sonia Garel (ENS, Paris) presented recent work on the developmental role of mammalian microglia and showed that both transient experimental depletion of microglia and prenatal inflammation result in supernumerary interneurons settling in cortical layers of the somatosensory barrel cortex. Both also lead to altered function of the cortical circuitry in offspring. This developmental role of microglia could be responsible for the observed effect of maternal inflammation during pregnancy on brain function of the child^17^.

Dr. Marco Prinz (University of Freiburg) provided an overview of the different resident myeloid cell types of the mammalian brain, which, aside from microglia, also include perivascular and meningeal macrophages. He reported his recent results on the postnatal proliferative behavior of microglia in normal and lesioned mouse brain, using a novel multicolor clonal analysis approach^18^. Under normal conditions, microglia are evenly spaced and proliferate very slowly. Following focal lesion of the facial nerve, there is a clonal expansion of microglia at the focus during a 4–8 week time interval; following that interval, microglia decline by actively migrating away from the scar and die by apoptosis.

The role of microglia in three major neurological diseases—Alzheimer’s disease, schizophrenia and glioblastoma—was presented by Dr. Helmut Kettenmann (Max Delbrück Center, Berlin), one of the founders and major contributors to the field of microglia biology. Microglia normally act as a focus in purinergic signaling, which plays an important role in brain function. The finding that decreased purinergic signaling, associated with microglia pathology, is part of the Alzheimer’s complex, offers a point of entry for a number of pharmacological approaches. Likewise, in the pathophysiology of schizophrenia, microglia may have an essential role through modulating synaptic plasticity. Finally, microglia represent a major cell type in gliomas, and were found to promote spreading of these cancers by secreting enzymes that facilitate tissue invasion. Active research is directed at unraveling the tumor cell-derived signals that reprogram microglia to act as promoters of tumor growth.

Finally, in the medicinal leech *Hirudo medicinalis* annelids, Dr. Christophe Lefebvre (University of Lille) showed that microglia are motile cells within the nerve tracts and neuropil^19^ (Fig. 1). These cells are functionally similar to vertebrate microglia as they migrate towards lesions, proliferate, and express proteins typical for macrophages in response to axon injury. Leech microglia release RNA-containing exosomes that engage in reciprocal interactions with neurons, thereby facilitating regeneration. Ongoing research focuses on establishing the transcriptome of activated microglia and exosomes in the leech. Interestingly, mammalian microglia are also known to use exosomes for interneuronal signaling^20^. The origin of the leech cells that are functionally related to microglia remains to be established.

Macroglial cells also heavily contribute to the function of the nervous system in physiological and pathological conditions, likely in concert with microglia. Dr. Nathalie Rouach (College de France, Paris) illustrated a new mechanism by which astrocytes regulate synaptic function. These findings may have a strong impact on medical research as, starting from an effect of the gap junction protein Connexin30 on LTP and behavior, she showed that in mice carrying Cx30 mutations, the glutamate uptake into astrocytes was enhanced, resulting in decreased glutamate concentration in the synaptic cleft^21^. This effect of Connexin is mediated via a previously unsuspected non-channel function, regulating astrocyte morphology and coverage of synapses. Interestingly, glial cells in *Drosophila* that are defective in a Connexin ortholog also display impaired neural function^22^.

A second example of functional conservation was provided by Dr. Christian Klämbt (University of Münster), who introduced his newly developed highly sensitive screens for larval behavioral abnormalities resulting from specifically impairing different subtypes of glia in *Drosophila*^23^. Dr. Klämbt focused on one gene, *shopper*, which encodes a sulfite oxidase suspected to be involved in glutamate homeostasis. Mutations in the human homolog (*SUOX*) result in neurological symptoms, including epileptic seizures. In *Drosophila*, *shopper* is specifically required in a glial subtype called ensheathing glia; similar to vertebrate astrocytes, ensheathing glia are involved in glutamate uptake and conversion to glutamine.

Dr. Alicia Hidalgo (University of Birmingham, UK) presented the genetic mechanisms underlying glial regeneration after CNS injury in *Drosophila*. Nerve injury in vertebrates is accompanied by the active proliferation of oligodendrocyte progenitors (OPCs), which requires the NG2 transmembrane protein, but how the transition from proliferating OPCs to differentiated oligodendrocytes is regulated is not understood. In *Drosophila*, CNS lesioning is followed by the proliferation of a specific glial subtype called neuropil glia^24^, which depends on the function of the fly ortholog of the NG2 gene, kon-tiki (kon). Kon is upregulated following wounding in a Notch pathway-dependent manner and promotes glial differentiation. The results support the conclusion that an evolutionarily conserved pathway drives glial proliferation followed by differentiation to restore glial integrity after injury.

Finally, the conserved role of glia in nervous system injury/repair was highlighted by a talk focusing on echinoderms. Dr. Jose Garcia- Arraras (University of Puerto Rico) introduced the regenerative function of radial glia cells in the sea cucumber^25^. These cells, which account for a major fraction of neural cells lining the radial cords, resemble vertebrate embryonic radial glia in structure and certain molecular components, such as intermediate filament proteins. In vertebrates, following lesioning of the radial cord, radial glia de-differentiate and become proliferative and migratory, thereby bridging the wound and allowing nerve fibers to regenerate.

## Concluding remarks

The debate on macro- and microglia development and function is wide open. The presentations discussed here illustrate the beneficial use of multidisciplinary approaches, from classic and molecular genetics to the newly developed technologies, including high-throughput analyses, bioinformatics and sophisticated imaging. This combination of approaches makes it now possible to tackle questions at different scales, from subcellular to organismal, and in many more organisms than the classic animal models. Filling these gaps will eventually provide a more comprehensive view on the connections existing between the immune and the nervous systems. Single-cell RNA sequencing, a promising new tool in reconstructing the evolutionary diversification of cell types, will help us understand how macro- and microglia evolved and acquired different functions, a division of labor that likely ensures more efficient responses in complex and long-lived organisms. Finally, dissecting the molecular and cellular networks involved in the immune response of the nervous system will greatly help in designing targeted therapeutic strategies that will eventually improve the quality of life.
